# Dementia and COVID-19: complications of managing a pandemic during another pandemic

**DOI:** 10.1590/1980-57642020dn14-040017

**Published:** 2020-12

**Authors:** Saeed Sadigh-Eteghad, Sepideh Seyedi Sahebari, Amirreza Naseri

**Affiliations:** 1 Neurosciences Research Center, Tabriz University of Medical Sciences – Tabriz, Iran.; 2 Student of the Research Committee, Tabriz University of Medical Sciences – Tabriz, Iran.

Dear Editor,

Dementia is a pandemic condition in aging societies, affecting about 50 million people.^1^ As the world faces the coronavirus disease 2019 (COVID-19) pandemic, dealing with a pandemic during another pandemic is a challenge. It is especially important when we know that aging is a well-established risk factor for dementia and also a risk factor for COVID-19 mortality.^2^

People with dementia may not be able to follow health recommendations such as keeping social distancing, covering their mouth and nose when coughing, and hand hygiene,^3^ which makes them more susceptible to getting infected. The clinical manifestation of COVID-19 may be different in people with dementia. A study consisted of 82 dementia patients reports a 67.1% rate of delirium as the most common symptom for COVID-19 infection, which was more than fever and dyspnea.^4^ As the management of patients with COVID-19 is mainly focused on preventing transmission, the lack of diagnosis in a huge number of infected people who cannot follow health recommendations can lead to further spreading of the virus. It can be more worrying when we know that a significant number of infected people are asymptomatic.^5^

Taking care of dementia patients is more probable to get a poor outcome.^6^ Recent studies found that individuals with dementia are more likely to have other COVID-19 risk factors such as diabetes and cardiovascular disease.^7^ Besides, a systematic review of literature found a twice as high pneumonia mortality rate in patients with dementia.^8^

During this pandemic, individuals with dementia showed higher mortality rate compared to patients without dementia which makes severe dementia independent risk factors for death due to COVID-19.^4^ ^,^ ^9^

According to statistics from the World Health Organization (WHO), 60–70% of dementia cases are in the form of Alzheimer disease (AD).^10^ The neuropsychiatric symptoms of AD, including depression, anxiety, apathy, agitation, and hallucinations, seem to worsen in COVID-19 confinement. Especially during prolonged hospitalization, when being away from the family, the familiar environment, and social and exercise groups may deteriorate patients’ dementia condition. In line with this, as the result of a study, among 38 AD patients with COVID-19 infection who were kept at home for nearly 2 months, 10 of them showed worsening of neuropsychiatric symptoms changes after confinement.^11^

The long-term effects of SARS-CoV2 infection on neurodegeneration are still unknown.

The direct neuroinvasive capacity of coronavirus to infect the central nervous system is not well known, but there are multiple possible mechanisms suggested for indirectly mediated inflammation.^12^ The cytokines produced in COVID-19 infection such as interleukin-1 (IL-1) and interleukin-6 (IL-6) may synergize with amyloid-stimulated type I interferon (IFN) in AD patients and play a role in the presentation of symptoms. This may be a silent impact of SARS-CoV2 on the deterioration of AD. It may also be a reason for facing severe COVID-19 symptoms earlier, after the onset of the infection in AD patients.^13^ ^,^ ^14^ Also, stress due to such a pandemic can accelerate cognitive decline.^15^

These findings may lead to an even higher rate of dementia in the long run after the COVID-19 pandemic.

Disruption in routine medical visits, diagnosis, and follow-up results in a rapid increase of severe cases of dementia in the future. We are not sure when the world society condition will become stable again; but we are sure that the mental and physical health of patients with dementia should not be neglected during this pandemic and COVID-19 should not overshadow dementia treatment.
